# FAIR adoption, assessment and challenges at UniProt

**DOI:** 10.1038/s41597-019-0180-9

**Published:** 2019-09-20

**Authors:** Leyla Garcia, Jerven Bolleman, Sebastien Gehant, Nicole Redaschi, Maria Martin, UniProt Consortium, Alex Bateman, Michele Magrane, Maria Martin, Sandra Orchard, Shriya Raj, Shadab Ahmad, Emanuele Alpi, Emily Bowler, Ramona Britto, Borisas Bursteinas, Hema Bye-A-Jee, Tunca Dogan, Leyla Garcia, Penelope Garmiri, George Georghiou, Leonardo Gonzales, Emma Hatton-Ellis, Alexandr Ignatchenko, Giuseppe Insana, Rizwan Ishtiaq, Vishal Joshi, Dushyanth Jyothi, Jie Luo, Yvonne Lussi, Alistair MacDougall, Mahdi Mahmoudy, Andrew Nightingale, Carla Oliveira, Joseph Onwubiko, Vivek Poddar, Sangya Pundir, Guoying Qi, Ahmet Rifaioglu, Daniel Rice, Rabie Saidi, Elena Speretta, Edward Turner, Nidhi Tyagi, Preethi Vasudev, Vladimir Volynkin, Kate Warner, Xavier Watkins, Rossana Zaru, Hermann Zellner, Alan Bridge, Lionel Breuza, Elisabeth Coudert, Damien Lieberherr, Ivo Pedruzzi, Sylvain Poux, Manuela Pruess, Nicole Redaschi, Lucila Aimo, Ghislaine Argoud-Puy, Andrea Auchincloss, Kristian Axelsen, Parit Bansal, Delphine Baratin, Teresa Batista Neto, Marie-Claude Blatter, Jerven Bolleman, Emmanuel Boutet, Cristina Casals-Casas, Beatrice Cuche, Edouard De Castro, Anne Estreicher, Livia Famiglietti, Marc Feuermann, Elisabeth Gasteiger, Sebastien Gehant, Vivienne Gerritsen, Arnaud Gos, Nadine Gruaz, Ursula Hinz, Chantal Hulo, Nevila Hyka-Nouspikel, Florence Jungo, Arnaud Kerhornou, Philippe Lemercier, Thierry Lombardot, Patrick Masson, Anne Morgat, Sandrine Pilbout, Monica Pozzato, Catherine Rivoire, Christian Sigrist, Shyamala Sundaram, Cathy Wu, Cecilia Arighi, Hongzhan Huang, Peter McGarvey, Darren Natale, Leslie Arminski, Chuming Chen, Yongxing Chen, John Garavelli, Kati Laiho, Karen Ross, C. R. Vinayaka, Qinghua Wang, Yuki Wang, Lai-Su Yeh, Jian Zhang

**Affiliations:** 3 Protein Information Resource, Georgetown University Medical Center, 3300 Whitehaven Street, NW Suite, 1200, 20007 USA; 4 Protein Information Resource, University of Delaware, 15 Innovation Way, Suite 205, Newark, DE 19711 USA; 1 European Molecular Biology Laboratory - European Bioinformatics Institute (EMBL-EBI), Wellcome Genome Campus, Hinxton, Cambridgeshire, CB10 1SD UK; 2 SIB Swiss Institute of Bioinformatics, Centre Medical Universitaire, 1 rue Michel Servet, 1211 Geneva 4, Switzerland

**Keywords:** Protein databases, Protein databases

## Abstract

UniProt continues to support the ongoing process of making scientific data FAIR. Here we contribute to this process with a FAIRness assessment of our UniProtKB dataset followed by a critical reflection on the challenges and future directions of the adoption and validation of the FAIR principles and metrics.

Data management and stewardship plans are nowadays essential to ensure the long-term sustainability of digital assets. The Findable, Accessible, Interoperable and Reusable (FAIR) principles^1^, first described in 2016, provide a framework defining the minimum elements required for good data management, making it easier for data providers to offer support for data driven knowledge discovery and innovation. Some of the main points of the FAIR principles address identification, licensing and data longevity policies.

Adopting the FAIR principles has proven to be a complex task that involves not only knowledge of your own data, but also awareness of metadata, schemata, protocols, policies, and community agreements. Another challenge lies in the vagueness of the original FAIR principles which offer a foundation layer for data management, but do not formally define how to fulfil the different elements under consideration. As a consequence, data providers may choose among a diversity of possible implementations making it difficult to critically assess the FAIRness of any resource. In order to overcome such limitations, a set of exemplar metrics were published in 2018^2^ and later complemented by a FAIR maturity framework^3^.

Although the importance of FAIR has been recognized widely by the research community via initiatives such as GO-FAIR (https://www.go-fair.org/) as well as a series of workshops to assess the FAIRness of current ELIXIR Core Data Resources (https://www.elixir-europe.org/platforms/data/fairness-core-resources), the adoption of the principles is still an ongoing process. Here we report our contribution to the process of FAIR adoption in the form of a FAIRness assessment on the Universal Protein Resource (UniProt)^4^. UniProt is a comprehensive resource for protein sequence and annotation data; it provides three main datasets: the UniProt Knowledgebase (UniProtKB), the UniProt Reference Clusters (UniRef) and the UniProt Archive (UniParc), all of them released every four weeks. UniProtKB is a central hub for the collection of functional information on proteins including accurate, consistent and rich annotation. UniRef provides clustered sets of sequences from UniProtKB and selected UniParc records. UniParc is a non-redundant dataset containing most of the publicly available protein sequences. With this FAIRness assessment, we aim to share our experience and the challenges we met with other resource providers and FAIR initiatives, so our experience can be used to further refine the FAIR principles and metrics.

## Our FAIRness Assessment Journey for UniProt

A FAIRness assessment for a large resource such as UniProt is not straight forward. UniProt data are published via a website (https://www.uniprot.org/uniprot) and distributed in multiple serialization formats, including a custom text format, XML, RDF/XML and FASTA. In addition, we also provide Application Programming Interfaces (API)s and File Transfer Protocol (FTP) downloads. The first question that we encountered during our FAIR assessment concerned this range of different distribution formats. Should all distributions be assessed as one or separately? Other resources that also support multiple serialization formats could face the same question when assessing their resources against the FAIR principles and metrics. In order to overcome these difficulties, ELIXIR Europe has supported a series of workshops to assess the FAIRness status of ELIXIR Core Data Resources (https://www.elixir-europe.org/platforms/data/fairness-core-resources). The resulting recommendation from one of these workshops was to use the FAIRest distribution. In the case of UniProt, this is its RDF/XML representation as this is based on well-structured vocabularies, i.e., schemata. This does, however raise the question: can a resource really be FAIR or just have FAIR representations? We do not know the answer yet, but more will be learnt as additional resources move to become FAIR with supporting FAIRness assessment.

We decided to use the UniProtKB dataset to assess the FAIRness of UniProt data because it is the most complex and most widely used of the three main UniProt datasets. We have followed the exemplar FAIR metrics^2^ together with supplementary information providing questions and assessments for other resources as reported by the FAIR maturity framework^3^. This assessment has been undertaken using UniProt release 2019_02. From one release to another, metadata such as dates and version together with the content itself are updated, but aspects such as identification schemata, access protocols and license usually remain the same. In the Online-only Table 1 we present our FAIR assessment results together with some supporting information.

Based on our assessment, UniProt is almost completely FAIR, with some remaining issues regarding the requirement for certification provided by a recognized authority. We cannot yet address these issues because it is currently unclear what a recognized authority, either FAIR or community based, would be for proteins.

## A Word on Identifiers, Metadata, and Data

The FAIR principles were designed for digital resources, their metadata and data. In order to relate a digital resource to their data content, there needs to be an explicit link between them. UniProt has an identifier as a dataset as a whole, “http://purl.uniprot.org/void#UniProtDataset”. Additionally, each set of data in UniProt, which we define as each UniProtKB entry in our assessment, also has an identifier, for example “https://purl.uniprot.org/uniprot/P05067”. Following the FAIR principles, all identifiers should be included in the respective metadata. From the dataset it should be possible to get to the content, i.e., UniProtKB entries in our case, or vice versa; whatever the chosen direction, dataset and content should be linked to each other. In the case of big datasets such as UniProt, the list of the entries contained in the dataset becomes too long to be included in the dataset metadata. A feasible alternative is to include a link from the entry to the dataset. If needed, a complete list of the dataset entries could be compiled by programmatic means, such as a SPARQL query designed to retrieve all entries included in dataset version 2019_02. Introducing a pattern-like link as part of the resource metadata would make it easier to reach its content. For validation purposes, an exemplar content identifier could also be included. This is a case that could be considered in the FAIR metrics. In the case of UniProtKB entries, such an identification pattern for content identifiers does exists and is documented in the Help pages (https://www.uniprot.org/help/accession_numbers).

In addition to the described link between resources and content, it is also important to take into account differences across multiple representations of a same dataset. In UniProt, the concept of an entry makes sense for our XML and custom text format, but it is hard to apply to the RDF world where each statement is an independent entity. For example, there are over 140 million UniProtKB entries in the 2019_02 dataset, but the corresponding RDF distribution also includes statements about many more International Nucleotide Sequence Database Collaboration (INSDC) “entries”, as well as over one billion other linked database “entries”. We also have to consider that most of our users do not want to retrieve what we consider to be a full dataset and will compose their own “subsets” via website or API queries, and we have therefore chosen to make each entry independently accessible.

Finally, the distinction between metadata and data is in many ways an arbitrary one. For some of our users the evidence for our assertions, e.g., publications, are metadata, while for other users they are critical data. Some serialization formats, especially those designed to be used by software tools, e.g. FASTA or GFF, make it impractical to include all data and metadata.

## Challenges and Evolution of FAIRness Assessments

We recognized the complexity that a large resource like UniProt poses for a FAIRness assessment. Even for smaller datasets FAIRness assessments are not a straightforward process. The current exemplar metrics, together with their question set are definitely a step forward in facilitating the FAIRification of resources; nonetheless, the process is still manual and requires human verification of the answers. Some of the questions such as those about schemata behind the identifiers and protocols, relate to third-party URLs, which are not necessarily in a machine-readable format. Information about HTTP or HTTPS can be found in Wikipedia, but would that be the correct URL for a FAIRness assessment? We do not know the answer and the metrics and questions do not help here. We mimicked the assessment examples provided as supplementary material at the GitHub FAIR metrics repository, as this seemed to be the simplest approach at this time.

The pilot project FAIRshake^5^ aims to make manual assessments easier. It presents users with a set of questions that are similar to those accompanying the exemplar metrics. The assessment process is still manual, based on questions and IRIs, and therefore presents the assessor with similar issues as does the question set accompanying the exemplar metrics. Rather than relying on manual assessments, the FAIR community should aim to create a semi or even fully automated validator to make assessments easier and comparable. Such a validator could, for instance, take account of the third-party URLs mentioned at the beginning of this section.

The FAIR principles and metrics are still evolving. They are gaining a momentum that should push digital resources to face the FAIR challenges and, by doing so, improve science. Communities will play an important role to make this a reality and the FAIR principles recognized this, for instance the principle F2 refers to rich metadata, R1 mentions a plurality of relevant attributes and R1.3 talks about community standards. Any FAIR validator should therefore be complemented with community-based validators. There are different accepted standards for datasets, e.g., DCAT (https://www.w3.org/TR/vocab-dcat/), EOSC-EDMI (https://eosc-edmi.github.io/) and Bioschemas^6^ (http://bioschemas.org/). For RDF distributions there is the external FAIR validator at YummyData^7^ (http://yummydata.org/) which strives to generate a computable FAIR metric. The data that we provide to YummyData are also used to improve our user documentation for the UniProt SPARQL endpoint at sparql.uniprot.org. This shows how being FAIR can also benefit the resource providers themselves.

While the FAIR principles and metrics cover a minimum of elements such as identifiers, license and provenance, community standards could go a step further by requiring additional metadata, thus improving interoperability and reusability. Despite their importance, data catalogs and datasets are not the only digital resources in existence. We expect that additional FAIR communities will emerge to adapt the existing principles to other digital resources such as training materials, software and services. The principles will then be tested outside their initial scope and adapted to add further exciting chapters to this FAIR tale.

## Online-only Table

**Online-only Table 1:** FAIRness assessment for UniProt.

Metric	Requirement	Resource	Resource data/content
	IRI for your resource	URL	PURL
		http://purl.uniprot.org/void#UniProtDataset	http://purl.uniprot.org/uniprot/P05067
		Release http://purl.uniprot.org/void#UniProtDataset_2019_02	
F1A	IRI for a registered identifier scheme for your resource’s IRI	PURL schema	PURL schema
		http://purlz.org	http://purlz.org
F1B	IRI to a document describing the persistence policy for the identifier of this data	http://purlz.org	http://purlz.org
			https://www.uniprot.org/help/accession_numbers
F2	IRI for machine-readable metadata for the resource	ftp://ftp.uniprot.org/pub/databases/uniprot/current_release/rdf/void.rdf	http://purl.uniprot.org/uniprot/P05067.rdf
	IRI to file format for this metadata	http://purl.uniprot.org/core/	http://purl.uniprot.org/core/
F3	Is the resource identifier specified in the metadata?	Yes	Yes
F4	URL to a search engine indexing your resource	https://www.google.com	https://www.google.com
	Search query/terms	First hit: uniprot	First hit: uniprot P05067
		First hit: universal protein resource	Sixth hit: amyloid beta precursor protein
A1.1	URL to the description of the protocol	HTTP	HTTP
		https://en.wikipedia.org/wiki/Hypertext_Transfer_Protocol	https://en.wikipedia.org/wiki/Hypertext_Transfer_Protocol
	Is the protocol open?	Yes	Yes
	Is the protocol (royalty) free?	Yes	Yes
A1.2	Is authorization required to access the content of your resource?	No	No
A2	URL to metadata longevity plan	ftp://ftp.uniprot.org/pub/databases/uniprot/previous_releases/README	Provided at dataset level
I1	URL to a specification language	RDFS and UniProt ontology	RDFS and UniProt ontology
		https://www.w3.org/TR/rdf-schema/	https://www.w3.org/TR/rdf-schema/
		http://purl.uniprot.org/core/	http://purl.uniprot.org/core/
I2	Maximum 3 IRIs for vocabularies used within the (meta) data	http://rdfs.org/ns/void#	http://purl.obolibrary.org/obo/GO
		http://purl.org/dc/terms/	http://purl.bioontology.org/ontology/OMIM/
		http://xmlns.com/foaf/0.1/	http://biohackathon.org/resource/faldo
I3	URL to a LinkSet (https://www.w3.org/TR/void/) for the resource	For the RDF/XML distribution	Provided at dataset level
		ftp://ftp.uniprot.org/pub/databases/uniprot/current_release/rdf/void.rdf	
R1.1	URL to license/terms of use for the resource	https://www.uniprot.org/help/license	Provided at dataset level
		https://creativecommons.org/licenses/by/4.0/	
R1.2	Maximum 3 IRIs used to describe the provenance of the resource	http://purl.org/dc/terms/	http://purl.org/dc/terms/
		http://xmlns.com/foaf/0.1/	http://purl.uniprot.org/core/
		http://purl.org/pav/	
	Maximum 3 IRIs used to describe domain information	Considering the dataset as domain here	Considering proteins as domain here
		http://purl.org/dc/terms/	http://purl.uniprot.org/core/
		http://xmlns.com/foaf/0.1/	http://purl.bioontology.org/ontology/OMIM/
		http://rdfs.org/ns/void#	http://purl.obolibrary.org/obo/ECO_
R1.3	IRI that represents certification from a recognized authority	http://yummydata.org/	Provided at dataset level
